# Radiofrequency Ablation Compared to Surgery for the Treatment of Benign Thyroid Nodules

**DOI:** 10.1155/2014/934595

**Published:** 2014-06-22

**Authors:** Stella Bernardi, Chiara Dobrinja, Bruno Fabris, Gabriele Bazzocchi, Nicoletta Sabato, Veronica Ulcigrai, Massimo Giacca, Enrica Barro, Nicolò De Manzini, Fulvio Stacul

**Affiliations:** ^1^UCO Medicina Clinica, Azienda Ospedaliero-Universitaria di Trieste, Cattinara Hospital, Strada di Fiume, 34100 Trieste, Italy; ^2^UCO Chirurgia Generale, Azienda Ospedaliero-Universitaria di Trieste, Cattinara Hospital, Strada di Fiume, 34100 Trieste, Italy; ^3^SC Radiologia, Azienda Ospedaliero-Universitaria di Trieste, Maggiore Hospital, Piazza dell'Ospitale, 34100 Trieste, Italy; ^4^UCO Radiologia, Azienda Ospedaliero-Universitaria di Trieste, Cattinara Hospital, Strada di Fiume, 34100 Trieste, Italy

## Abstract

*Objective*. Benign thyroid nodules are a common occurrence whose only remedy, in case of symptoms, has always been surgery until the advent of new techniques, such as radiofrequency ablation (RFA). This study aimed at evaluating RFA efficacy, tolerability, and costs and comparing them to hemithyroidectomy for the treatment of benign thyroid nodules. *Design and Methods*. 37 patients who underwent RFA were retrospectively compared to 74 patients surgically treated, either in a standard inpatient or in a short-stay surgical regimen. Efficacy, tolerability, and costs were compared. The contribution of final pathology was also taken into account. *Results*. RFA reduced nodular volume by 70% after 12 months and it was an effective method for treating nodule-related clinical problems, but it was not as effective as surgery for the treatment of hot nodules. RFA and surgery were both safe, although RFA had less complications and pain was rare. RFA costed €1,661.50, surgery costed €4,556.30, and short-stay surgery costed €4,139.40 per patient. RFA, however, did not allow for any pathologic analysis of the nodules, which, in 6 patients who had undergone surgery (8%), revealed that the nodules harboured malignant cells. *Conclusions*. RFA might transform our approach to benign thyroid nodules.

## 1. Introduction

Thyroid nodules are an extremely common occurrence and their prevalence in the general population is estimated to range between 50% and 67%, according to autoptic [1] and ultrasonographic [2] data. Until recently, though, only a few remedies have been available for such a common disease. According to current guidelines [3], benign thyroid nodules should be followed up clinically. In order to avoid nodule enlargement, levothyroxine therapy, whose efficacy is still limited [4], and/or iodine should be taken into account in young patients with small nodules living in iodine-deficient areas. However, if nodular growth causes the onset of symptoms or cosmetic concerns the only available remedy so far has been surgery. Not only is curative surgery invasive but it also has several drawbacks [5]. Therefore, new treatment modalities for such a common disease are needed.

Radiofrequency ablation (RFA) is a relatively new technique that seems a safe and effective method for the debulking of large benign thyroid nodules [6] and a promising treatment modality for autonomously functioning nodules [7–11]. Nevertheless, guidelines are cautious and RFA is not recommended yet in the routine management of benign thyroid nodules [3, 4].

Here we aimed at (i) evaluating RFA efficacy, tolerability, and costs and (ii) comparing RFA outcomes to those of hemithyroidectomy, including short-stay thyroid surgery, for the treatment of benign thyroid nodules.

## 2. Materials and Methods

### 2.1. Patient Selection and Study Design

#### 2.1.1. Patient Selection

To assess RFA efficacy, complications, and costs we followed 37 patients who underwent RFA of their thyroid nodules between March 2012 and May 2013. Overall, 38 RFA have been performed in 37 patients, as in one case the treatment was repeated. Before the procedure, nodules were evaluated by fine needle aspiration biopsy (FNAB) twice and classified according to the British and Italian reporting systems for thyroid cytopathology [12]. RFA was performed on benign solitary nodules (diagnostic category Thy2/Tir2) that measured >2 cm and caused clinical problems (neck pain, voice change, foreign body sensation, discomfort and cough, and cosmetic concerns) or problems related to thyrotoxicosis, according to current recommendations [6]. Since caution should be exercised in patients with contralateral vocal cord palsy [6], patients presenting with voice changes underwent an otolaryngological visit and laryngoscopy to exclude such injury.

To compare RFA outcomes with surgery, we selected 74 patients among 525 patients who underwent thyroid surgery from May 2005 to May 2013. These were all patients with benign single thyroid non-autoimmune nodules, who had undergone hemithyroidectomy, performed either in a standard inpatient surgical regimen (>24 hours, *n* = 64, hEMItx) or in a short-stay regimen (<24 hours, *n* = 10, short-stay). Patients who had already undergone contralateral thyroid lobectomy or who had had cytological preoperative diagnosis of malignancy (diagnostic category Thy5/Tir5) or suspected malignancy (Thy4/Tir4) as well as patients with nodules of undetermined significance (Thy3/Tir3) and/or with increased calcitonin levels were excluded from the study.

This work was approved by the Ethical Committee of the Azienda Ospedaliero Universitaria di Trieste and informed consent was obtained from each patient, after full explanation of the purpose and nature of all procedures used, before inclusion in the study.

#### 2.1.2. Study Design

This study aimed at evaluating RFA outcomes and at comparing them to those of surgery. Firstly, to assess RFA efficacy and tolerability, patients underwent a medical visit, ultrasonography (US), and thyroid-stimulating hormone (TSH) measurement at baseline, 1, 3, 6, and 12 months after the procedure. During these visits, medication, nodule-related symptoms (yes/no), cosmetic score (1 = no palpable mass; 2 = palpable mass; 3 = mass visible on swallowing; 4 = easily visible mass), cosmetic results (poor, acceptable, good, and excellent), pain, and/or other complications (hematomas, skin burns, fever, and voice change) were recorded. To measure nodule volume (*V*) and volume reduction (*V*reduction) the following formulas were used: *V* = *πabc*/6 (where *V* is the volume,* a* is the maximum diameter, and* b* and *c* are the other two perpendicular diameters) and (*V*reduction = (initial − final*V*/initial*V*)∗100). Therapeutic success was defined as a volume reduction >50% [6]. Vascularity was evaluated according to a 4-point scale where 0 is defined as no visible flow, 1 as peripheral flow only, 2 as peripheral flow with a small amount of central flow, 3 as peripheral flow plus extensive intranodular flow, and 4 as central flow only.

Then, to compare the efficacy and tolerability of RFA to surgery, we took patient history, any current complaints (neck symptoms yes/no), cosmetic results (graded as poor, acceptable, good, and excellent), drug history, and TSH at baseline and one year after surgery. Pain and complications of surgery were analysed retrospectively from the medical records.

The endpoints for efficacy were (i) cure of nodule-related symptoms, (ii) patient satisfaction with cosmetic results, and (iii) antithyroid drugs (ATD) withdrawal. Tolerability was expressed as (i) cases of postoperative pain, (ii) cases of iatrogenic hypothyroidism, and (iii) number of complications. Cost analysis is discussed below. The contribution of final pathology to patient management was also analysed and discussed.

### 2.2. Radiofrequency Ablation

**Figure 1:**
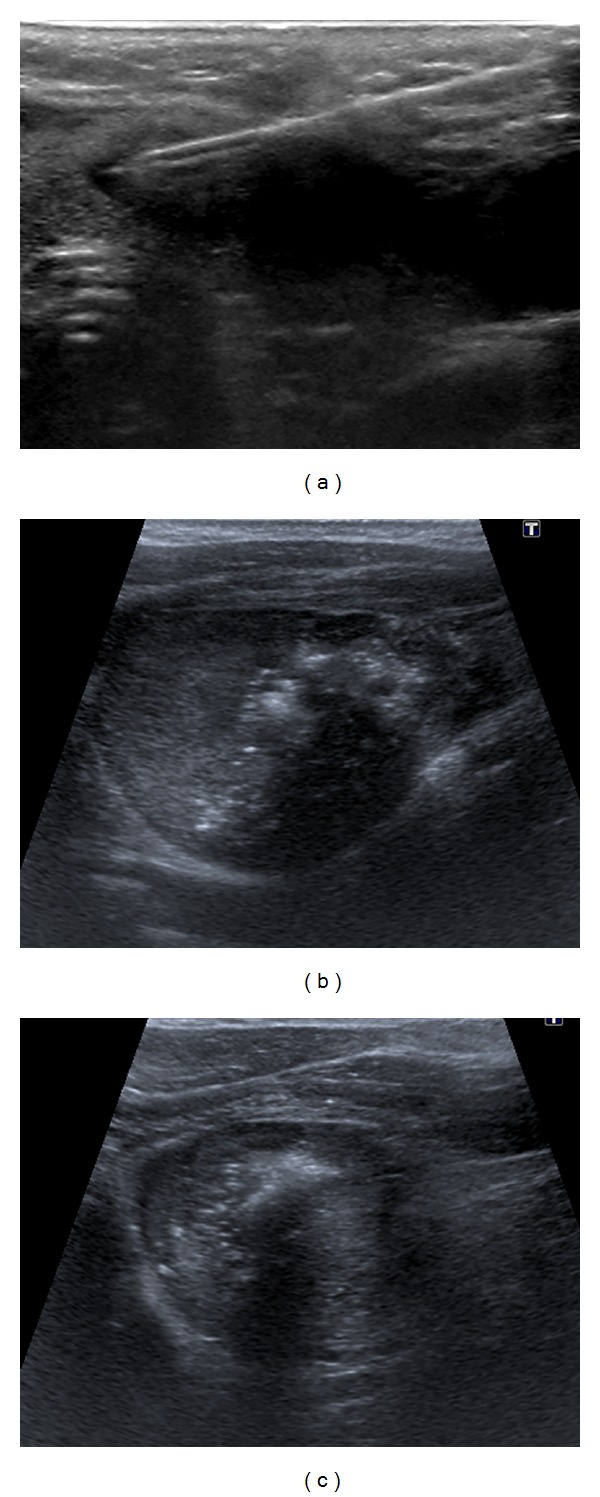
(a) Transverse US image shows the transisthmic approach. The probe, whose active part is placed in the nodule through its isthmus, is inserted from the medial to the lateral part of the nodule and is visible along its longitudinal axis. (b-c) Transverse US images show the moving-shot technique. RFA is performed unit by unit, aiming at ablating all the subunits of the nodule, which turn into microbubbles (hyperechoic areas with posterior barrage of the ultrasound beam). Initially, (a) the probe tip is positioned in the medial and deepest part of the nodule and subsequently (b-c) in its most superficial and lateral parts.

RFA was performed in an outpatient regimen by a well-trained radiologist experienced in US, FNAB, and RFA procedures. Before ablation, an intravenous access was obtained via an antecubital vein. Patients were placed in a supine position with their neck extended. RFA did not require general anesthesia, and our patients underwent only local anesthesia at the puncture site with 2% lidocaine hydrochloride. We used a radiofrequency generator (VIVA RF generator; AMICA RF generator) and 18-gauge internally cooled electrodes with a 0.7–1.0–1.5 cm active-tip (star RF probes STARmed; Amica probes, HS AMICA). The electrode was inserted into the thyroid nodule with a transisthmic approach [13] under US-guidance by using a 7–16 MHz linear probe on a real-time US system (Aplio XG, Toshiba Medical Systems Corp.), as shown in Figure 1(a). To perform RFA the moving-shot technique was used [13]. Ablation was begun with 30 W, starting from the deepest areas of the nodule. If a hyperechoic zone had not formed at the electrode tip within 5–10 seconds, radiofrequency power was progressively increased in 10 W steps up to 60 W. The power would have been reduced or turned off in cases where patients could not tolerate pain during ablation, but no such case was encountered in our cohort. Moreover, during the procedure there was an anaesthetist to assist the radiologist in case of pain [6], vasovagal reactions [6], or interference with pacemaker devices [14], but no such case was encountered in our cohort. Ablation was terminated when transient hyperechoic zones could be identified around the whole nodule, as shown in Figures 1(b)-1(c). At the end of the procedure a mild compression was applied to the site of the needle insertion for 5–10 minutes and patients remained under observation for a few hours in case they had complications. Pain was recorded 4 hours after the procedure and scored according to the visual analogue scale (VAS) ranging from 0 to 10 with the words “no pain = 0” on the left hand side and “worst possible pain = 10” on the right hand side.

### 2.3. Hemithyroidectomy: Surgical Technique

Patients were allocated to short-stay hemithyroidectomy according to their age, residence, nodule size, and comorbidities [15]. All operations were carried out by the same team, which included two surgeons (lead and assistant surgeon). Hemithyroidectomy was performed by a single access of 2–5 cm in the middle area of the neck, approximately 2 cm above the sternal notch. Patients, under general anaesthesia, were placed in the supine position with the neck extended. Minimal subplatysmal flaps were created. The midline was incised and the strap muscles were retracted laterally. The vessels of the upper thyroid peduncle were selectively ligated or closed by conventional vascular clips. Special care was taken to preserve the parathyroid glands and the inferior laryngeal nerve. Once the thyroid lobe and isthmus had been removed, the area was examined for bleeding. If there was no bleeding, the incision was closed with sutures. In some cases, a surgical drain was placed to remove fluids from the area in the following days. Anaesthesia was discontinued and medication was given to wake the patient. In those patients who underwent standard inpatient hemithyroidectomy, postoperative pain and serum calcium were measured 4 and 24 hours after surgery, together with parathormone (PTH). Those who underwent short-stay surgery were admitted the day of operation and observed overnight. Here postoperative pain and serum calcium were evaluated 4 and 20 hours after surgery, together with PTH. All the patients underwent laryngoscopy after the procedure. Discharge criteria included no wound or airway problems, stable vital signs, tolerance of normal diet and activity, and an upsloping serum calcium curve.

### 2.4. Cost Analysis

Cost analysis included the procedure (RFA or surgery) and the respective pre- and postprocedural exams.

#### 2.4.1. RFA Costs

Preprocedural exams included laboratory tests (full blood count, coagulation tests, TSH, thyroid hormones, calcitonin, and thyroid autoantibodies), US and anaesthesiological visit, and an otolaryngological visit in selected cases (among our patients, 2 out of 37 underwent such a visit). The cost of the procedure included the equipment, consisting of scanner, needle, and drugs, as well as the personnel, consisting of one radiologist, one anaesthetist, and two nurses. In particular, the cost of any US scanner can be divided into depreciation and maintenance. Apart from maintenance, which is negligible, the purchase cost of our US scanner was €97,489, which equals an average annual cost of €12,186 over 8 years of depreciation. Therefore, assuming 250 working days per year, the daily cost of the scanner is €48.74 per day and since it is used for 12 hours per day and the length of one RFA is 45 minutes, the final cost of a scanner is €3.04 per exam. Drugs were lidocaine (€0.013/mL), midazolam (€0.099/mL), fentanyl (€0.140/mL), droperidol (€3.168/mL), and saline (€0.834/mL). The cost of the radiologist and the anaesthetist was calculated according to their working time, which is 1,330 hours/year, and their gross annual salary, which is €101,040 in both cases (excluding social security and termination bonus), corresponding to a salary of €76 per hour. Likewise, the gross annual salary of a nurse is €39,174 (excluding social security and termination bonus), corresponding to a salary of €29 per hour. Postprocedural exams included TSH measurement and a US scan.

#### 2.4.2. Surgical Costs

Preprocedural exams included laboratory tests (including full blood count, urea and electrolytes, glucose, urine analysis, liver function test, coagulation blood tests, blood type, HIV and markers of hepatitis, and TSH), ECG, chest X-ray, and otolaryngological and anaesthesiological visits. The costs of the procedure included hospital stay (€375/night), the operating theatre (€24/min), the variable costs for technical textiles (€31.50), the sterilization of surgical instruments (€95.30), scalpels (€0.20), suction cannulas (€23.80), sutures (€23.99), bipolar forceps (€19.05), tampons (€0.69), and Redon sets (€3.04). The drugs that were used were remifentanil (€304.20/5 mg) and desflurane (€0.35/mL). Personnel consisted of two surgeons, one theatre nurse, two nurses, and one anaesthetist, whose costs were calculated as above. Postprocedural exams were laboratory tests (calcium, PTH) and an otolaryngological visit.

### 2.5. Statistical Analysis

Continuous variables were evaluated by ANOVA. *χ* ^2^ test was used to compare sex, nodules' characteristics, symptoms, hyperthyroidism, and autoimmunity between the two groups. The criterion for statistical significance was *P* < 0.05. All analyses were conducted using SAS (SAS Institute Inc., Cary, NC, USA).

## 3. Results

**Figure 2:**
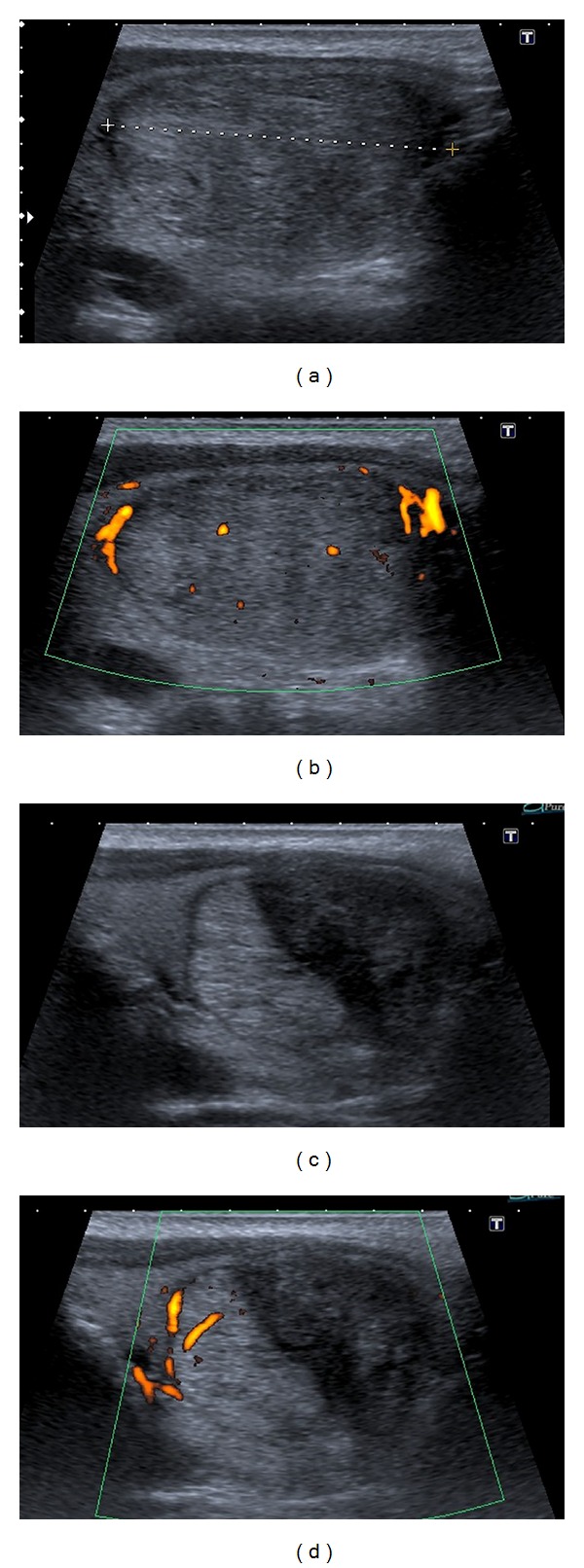
Longitudinal B-mode and power Doppler US images, obtained before (a-b) and after RFA (c-d), show the effects of the procedure at 1 month. The danger triangle, which remains undertreated, is clearly visible on the medial side of the nodule (c-d). Apart from the overall reduction in size, the treated area of the nodule appears hypoechoic and avascular (c-d).

**Figure 3:**
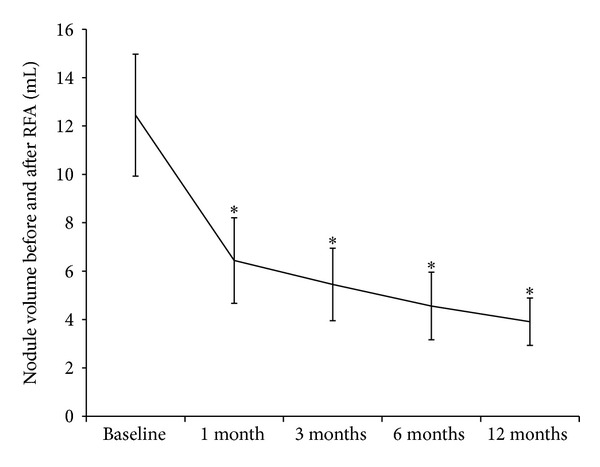
Volume (mL) reduction at 1, 3, 6, and 12 months after RFA. Data are expressed as mean ± SEM. *P < 0.05 versus baseline.

The characteristics of the thyroid nodules are reported in Table 1. There were no differences between the nodules treated by RFA and those treated by surgery in terms of cystic component, macrocalcifications, and vascularization. In this study, RFA significantly reduced thyroid nodule volumes as shown in Figure 2. In particular, the nodules' volume was 12.45 ± 2.52 mL at baseline, 6.44 mL ± 1.77 mL after 1 month from the procedure (*P* < 0.05 versus baseline), 5.45 ± 1.50 mL after 3 months (*P* < 0.05 versus baseline), 4.56 ± 1.40 mL after 6 months (*P* < 0.05 versus baseline), and 3.91 ± 0.98 mL after 12 months (*P* < 0.05 versus baseline). In other words, the volume decreased by 47%, 61%, 66%, and 70% at 1, 3, 6, and 12 months after RFA. The greatest volume reduction was observed within the first month and this was more pronounced in the lesions with more than 50% of liquid content. Therapeutic success was achieved with a single session in 36 out of 37 patients, as only one patient required two sessions to achieve a final volume reduction of 77%. This patient had a solid nodule, with peripheral plus intranodular flow, measuring 42 × 36 × 31 mm. In this case, RFA was repeated 6 months after the first session because the nodule had decreased less than 50% of its initial volume. Apart from volume reduction, RFA lessened nodular echogenicity and made intranodular vascularity disappear, as shown in Figure 3. On the other hand, hemithyroidectomy was successfully completed in all the cases. In particular, among the 74 patients who underwent surgery, 64 subjects underwent a hemithyroidectomy performed in a standard inpatient surgical regimen and 10 subjects underwent a hemithyroidectomy performed in a short-stay surgical regimen.

Patients' characteristics at baseline are reported in Table 1. The groups differed only in terms of number of patients with neck symptoms, which was higher in the surgical group. Focusing on the efficacy of these techniques, RFA was able to cure nodule-related symptoms in the majority of patients (11 patients out of 13) and was not inferior to surgery, as shown in Table 2. In particular, while neck symptoms were resolved completely in 11 patients out of 13 treated by RFA, they ameliorated in the other 2 cases, as these patients were suffering from gastroesophageal reflux disease, which might have contributed to their local discomfort. Secondly, there were no differences between RFA and surgery in terms of cosmetic results, which were excellent for the vast majority of patients in both groups (35 out of 37 patients for RFA and 65 out of 74 for surgery). In particular, RFA significantly reduced the cosmetic score, which went down from 3.4 to 2.4 per patient (*P* < 0.05); in other words, nodules that were visible could no longer be seen after the procedure. Cosmetic results were graded as “excellent” by 35 patients (94%), while the remaining two had nodules measuring more than 35 mL initially which resulted in a final volume of 15 mL. In these two cases, cosmetic results were scored as “acceptable.” On the other hand, 88% of the patients (65) who had undergone surgery judged the results as “excellent,” while they were “good” for 9% of the patients (7) and “acceptable” only for 3% of the patients (2), depending on their scar. In particular, in our cohort, the cosmetic benefit of thyroid nodule removal was felt to outweigh the presence of a scar in 65 patients out of 74, while in the remaining 9 patients the scar compromised the final cosmetic result [16].

Overall, the volume reduction achieved by RFA did not affect thyroid function, which remained unchanged throughout the entire follow-up period. In particular, TSH was 1.30 ± 0.25 *μ*U/mL at baseline, 1.48 ± 0.25 *μ*U/mL at 1 month, 1.24 ± 0.22 *μ*U/mL at 3 months, 0.95 ± 0.15 *μ*U/mL at 6 months, and 1.37 ± 0.28 *μ*U/mL at 12 months after the procedure. Having said that, in the subgroup of hyperthyroid patients (*n* = 12), RFA improved thyroid function as TSH increased from 0.28 ± 0.08 microU/mL to 1.35 ± 0.31 microU/mL (*P* < 0.05) after just one month from the procedure and it was maintained throughout the study period. Thanks to this effect, 4 patients out of 12 could stop or avoid taking any ATD, as their thyroid function normalized completely, whereas the remaining 8 patients could reduce the number of tablets from 1 tablet every day to 1 tablet every two days. Nevertheless, this effect was not enough for these 8 patients and RFA was significantly less effective compared to surgery for the treatment of autonomously functioning nodules, as shown in Table 2.

RFA was extremely well tolerated. Postoperative pain was rare as in only 2 cases out of 38 sessions pain was reported. Pain was mild, was treated by paracetamol, scored 2.5 ± 0.8 points, and ceased within 2 weeks. Apart from this, in our study, RFA complications were 1 case of transient voice change and 1 case of late-onset, painless thyroiditis with transient thyrotoxicosis. The voice change was treated by prednisone and was resolved within 1 and a half months. The late-onset painless thyroiditis with thyrotoxicosis developed 3 months after the procedure and resolved spontaneously within 30 days. Moreover, such thyroiditis did not cause hypothyroidism. There were no vasovagal reactions or cases of haemorrhage, skin burn, or fever. Surgery was also well tolerated, but not as much as RFA (see Table 2). Pain was always treated by paracetamol postoperatively so it was mild and scored on average 2.9 ± 0.1. Surgical complications were only transient and resolved within 6 months. These included 4 cases of transient hypocalcemia (serum calcium < 8.5 mg/dL with slightly reduced but normal PTH), 6 cases of monolateral transient nerve palsy, and 2 cases of wound complications. There were no cases of bleeding requiring reintervention. Only 17 patents had to take levothyroxine after surgery for the first time. Considering that 6 patients had been taking levothyroxine before surgery, the majority (51) of the remaining 68 patients, however, remained euthyroid.

Focusing on costs, the length of one RFA session was 45 minutes and it was performed in an outpatient regimen and cost of €1,661.50. The mean operative time for hemithyroidectomies performed in a standard inpatient regimen was 80 minutes, the length of the hospital stay was 2.33 days, and their mean cost was €4,556.30. In the group requiring short-stay surgery, mean operative time was 82.5 minutes, hospital stay was 1 day, and the cost was €4,139.40, as reported in Table 3.

Having said that, another aspect we considered was the contribution to patient management of final pathology, which only occurred in the surgical approach. Final pathology showed that 68 patients out of 74 (92%) presented with benign thyroid nodules. 44 nodules were follicular adenomas, 12 were nodular thyroiditis, 6 were colloid lesions, 4 were degenerative cysts, 1 was a Hurtle cell adenoma, and 1 was a nodule within Riedel's thyroiditis. So, despite the fact that before surgery all FNAB were benign, 6 nodules (8%) were found to be harbouring malignant cells on final pathology and 5 cases were papillary microcarcinomas (<1 cm) while 1 case was a papillary carcinoma (>1 cm).

## 4. Discussion

Our comparative data show that RFA significantly reduces thyroid nodule volume and relieves from nodule-related clinical problems, such as local symptoms and cosmetic concern, as effectively as surgery. In this study, 12 months after RFA, thyroid nodules had shrunk by 70%, and the best results were achieved in nodules with a mixed content and an initial volume <35 mL. This is in line with previous studies demonstrating that RFA can reduce nodule volume by 33–58% after one month, by 51–85% after six months [11, 17], and by 93% after four years [18] from the procedure. Different percentages of volume reduction can be attributed to the number of sessions, which in our study were 1.03 per patient, whereas in other studies were 1.8 per patient [18]. Moreover, although the length of our follow-up is 12 months, it has already been demonstrated that the volume reduction achieved is maintained up to 4 years after RFA. Volume reduction did not affect thyroid function in euthyroid patients, which might have been due to the fact that there was a negligible number of autoimmune thyroiditis [19], but it was able to normalize TSH in some of the patients with hot nodules. However, hyperthyroidism was completely resolved only in 33% of patients, indicating that in our study RFA was significantly less effective than surgery for the treatment of hyperfunctioning nodules. This is consistent with what has already been reported by the groups of Deandrea et al. [11], who showed that hyperfunction was fully controlled in 24% of patients at 6 months after RFA, and by Faggiano et al. [7], who further showed that hyperfunction was fully controlled in 40% of patients with hot nodules at 12 months after RFA. Having said that, Spiezia [10] and colleagues have demonstrated that 2 years after RFA thyroid function normalized in 100% of patients with pretoxic nodules and in 53% of patients with toxic nodules. Further studies are needed to clarify whether two RFA sessions could cure hyperfunctioning nodules as effectively as surgery, since incomplete recovery could be explained by the difficulty in ablating the entire nodule and the regrowth of the untreated peripheral portion [9]. So, our comparative study (RFA versus surgery) shows that current recommendations on RFA [6] are effective and applicable in treating thyroid nodules causing symptoms and cosmetic concerns, while the best approach to treat autonomously functioning nodules is surgery and not RFA.

In general, consistent with what has been reported by Lim et al. [18] RFA was safe and extremely well tolerated. Postoperative pain was rare. Apart from two cases of mild pain and one case of a transient voice change there were no other complications, which could be ascribed to the technique that was used. To avoid thermal nerve injury the moving-shot technique and undertreating the area adjacent to the nerve [13], also called the danger triangle, as shown in Figure 2, have been suggested. In our study, one patient developed a late-onset, painless thyroiditis with transient thyrotoxicosis 3 months after RFA, which we report for the first time as one of the potential RFA complications. The presence of a thyrotoxicosis was suggested by thyroid hormones' increase and TSH decrease. The presence of a thyroiditis was suggested by the positivity of anti-TPO antibodies, which were absent at baseline, and the suppressed thyroid radioiodine uptake [20]. Such thyroiditis with thyrotoxicosis was transient, as it resolved spontaneously within a few weeks and it did not cause hypothyroidism. This case of painless thyroiditis with thyrotoxicosis brings into mind postaspiration thyrotoxicosis, which is believed to be an inflammatory process that takes place after needle aspiration of the thyroid and triggers the release of thyroid hormones [21]. Given the proximity to RFA, it is conceivable that such thyroiditis with thyrotoxicosis might have been caused by this procedure through a mechanism similar to that of postaspiration thyrotoxicosis [21]. As compared to RFA, surgery caused hypothyroidism in 25% of patients and had a higher complication rate, although they were all mostly mild and transient, and nobody developed life-threatening problems such as haemorrhage, laryngeal edema, or tetany, in keeping with previous data [22, 23]. Overall, there were 6 cases of transient nerve palsy, 4 cases of transient hypocalcemia, and 2 cases of wound complications. Hypocalcemia after hemithyroidectomy might be explained by the unintended excision of one or more parathyroids together with metabolic factors such as “hungry bones” associated with thyrotoxicosis [22]. Although the majority of patients were very pleased with surgical results, consistent with the literature [24], our data on the baseline characteristics might suggest that patients are not always comfortable with undergoing surgery. In fact, in our view, only the onset of neck symptoms or strong cosmetic concerns, which outweigh the lingering apprehension over the risk of surgical complications, would have encouraged our patients to undergo surgery.

The last decades have witnessed a quiet revolution in relation to the management of benign thyroid diseases [25]. Advances in surgical techniques are making surgery not only safer but also minimally invasive if not totally scarless. In addition, thanks to the improvements in the management of bleeding, pain, and hypoparathyroidism, hospital stays have decreased to below 23 hours [26]. Today, the advantages of outpatient surgery include reduced costs, reduced inpatient waiting lists, increased availability of inpatient beds, and the psychological benefit of avoiding prolonged hospitalization. Having said that, there are clearly social and clinical circumstances in which outpatient thyroidectomy is not possible [15]. Moreover, even if it were feasible, outpatient thyroid surgery remains controversial, given that the likelihood of delayed haemorrhage and one case of death have been reported [27]. Notwithstanding several potential benefits, those who argue against outpatient surgery maintain that hospital cost savings should not be at the expense of patient safety. In regard to this point, it is abundantly clear that RFA is safer. There is no risk of hypocalcemia, nerve injury can be easily avoided (voice changes are reported in 1% of cases), and there have been no reports so far of life-threatening events [6, 13, 18, 19]. In addition, RFA does not require general anaesthesia. So, RFA could prove useful for treating weak or elderly patients or those with challenging hemostasis, comorbidities, and other clinical or social issues not conducive to surgery. In addition to its efficacy and safety, here we show that RFA compares extremely favorably to surgery in terms of costs, as it costs roughly 2.6 times less than surgery, without including the fact that sick-leave is significantly shorter and social costs are significantly lower. However dramatically renewed surgery might be, the greatest shift in the future routine management of benign thyroid nodules will probably be determined by the availability of nonsurgical, minimally invasive techniques. These techniques together with recent advances in molecular biology are likely to minimize unnecessary surgery [28].

Previous studies have reported that malignancy can be found within a benign nodule. The group of Arora and colleagues analysed 826 thyroid surgical specimens and came upon 8 carcinomas (2%) within benign nodules [29]. In another work, Park and colleagues discovered occult papillary carcinomas in 9.2% of adenomatous goiters and 4.3% of follicular adenomas [30]. This is in line with the recent study carried out by Wang and collaborators who showed that on average 6% of benign nodules are found postoperatively malignant by cytopathology diagnosis [31]. These findings clearly demonstrate that some benign nodules may harbour microscopic foci of malignancy that can be missed at the FNAB due to sampling error. It is not clear when these foci developed, whether they are bound to grow, and whether molecular biology will enable us to overcome this issue by differentiating these nodules from those that are totally benign [32]. Here we found 6 carcinomas in 74 nodules (8%) that had been classified as benign nodules by FNAB. The majority of them (5 out of 6) were microcarcinomas. It has to be noted, though, that before surgery FNAB had not always been performed with ultrasonography. It is exactly for reducing the likelihood of false cytologically benign FNAB that before RFA it is recommended [6] to perform two ultrasonographic-guided FNAB, in both functioning and nonfunctioning nodules, as we did in this study before RFA.

It is absolutely clear that the strength of surgery is that it allows for the final pathology and therefore the diagnosis and cure of cancer. However, it is argued that low-risk thyroid cancer, such as microcarcinomas, is being overtreated nowadays [33] as small papillary cancers may never progress to cause symptoms or death. To back this view a recent observation trial on papillary thyroid microcarcinoma has in fact shown that tumor enlargement and new onset of node metastases were detected in only 6.5% and 1.4% of patients after 5 years of observation [34]. Moreover, none of the patients having a microcarcinoma who underwent surgery after 5 years of follow-up showed postoperative carcinoma recurrence and none of the patients who underwent observation showed distant metastasis or died of thyroid carcinoma. These data would therefore suggest that papillary microcarcinoma has an indolent nature and immediate surgery is not mandatory [35]. Moreover, a recent study by the group of Valcavi has demonstrated that percutaneous laser ablation is technically feasible for complete destruction of papillary microcarcinomas [36]. Still, one of the biggest issues about the use of RFA on thyroid nodules is that we still do not know its impact on malignancy in the long term. Although RFA has been successfully used for locoregional control of cancer or improvement of cancer-related clinical symptoms in patients with recurrent thyroid cancer and either a high surgical risk or unwillingness to undergo repeated surgery, there are no long-term follow-up data on radiofrequency ablated benign thyroid nodules harbouring malignant cells and therefore on how to monitor these cases. It is exactly for this reason that it would be sensible to continue the monitoring of the ablated nodules at least yearly for 5 years. Having said that, if RFA could cure occult carcinomas, then it would definitely kill three birds (efficacy, safety, and costs) with one stone.

## 5. Conclusion

In conclusion, our comparative study suggests that RFA represents an effective alternative to surgery, which is very expensive and occasionally unnecessary, for the treatment of benign thyroid nodules causing local symptoms or cosmetic concerns. Nevertheless, this study does not dismiss surgery, which, on the contrary, is more effective than one RFA session for treating nodules with an initial volume greater than 35 mL as well as for autonomously functioning nodules. Since RFA does not allow for final pathology, patients should undergo two ultrasonographic-guided FNAB prior to the procedure and should be followed at least yearly for five years.

## Figures and Tables

**Table 1:** Patients' characteristics at baseline before either RFA or surgery.

Characteristics	RFA (*n* = 37)	Surgery (*n* = 74)	*P* value
Age (years)	58.3 ± 3.6	54.9 ± 3.4	n.s.
Sex (M/F)	12/25	17/57	n.s.
Nodule maximum *Ø* (mm)	35.7 ± 2.5	38.8 ± 3.2	n.s.
Nodule volume (mL)	12.4 ± 2.5	12.8 ± 2.1	n.s.
Solid nodules (Y/N)	28/9	43/31	n.s.
Vascularity	2.1 ± 0.1	2.1 ± 0.2	n.s.
Macrocalcifications (Y/N)	8/29	19/55	n.s.
Patients with symptoms (Y/N)	13/24	43/31∗	0.03
Hyperthyroidism (Y/N)	12/25	20/54	n.s.
TSH (microU/mL)	1.2 ± 0.2	1.3 ± 1.9	n.s.
Calcitonin (pg/mL)	1.8 ± 0.3	2.8 ± 0.5	n.s.
Anti-TPO and/or anti-TG Ab (Y/N)	3/34	7/67	n.s.

Results are expressed as mean ± SEM. Anti-TPO Ab, anti-thyroperoxidase antibodies; anti-TG Ab, anti-thyroglobulin antibodies; n.s., nonsignificant; RFA, radiofrequency ablation; TSH, thyroid-stimulating hormone; Y/N, yes/no. Solid nodules had less than 50% of cystic component.

**Table 2:** Efficacy and tolerability of RFA compared to surgery.

Outcomes	RFA (=37)	Surgery (=74)
Efficacy		
Patients with symptoms	13	43
**Resolution of nodule-related symptoms**	**11**	**43**
Patients with hyperfunctioning nodules	12	20
**ATD withdrawal/thyroid function normalization**	**4**	20*
Patients with cosmetic concerns	37	74
**Excellent cosmetic results**	**35**	**65**

Outcomes	RFA (=37)	Surgery (=74)

Tolerability		
Patients without levothyroxine prior to treatment	31	68
**Hypothyroidism**	**0**	17*
Total number of procedures	38	74
**Postoperative pain**	**2**	74*
**Complication rate**	**2**	10*

*P < 0.05 versus RFA. ATD, antithyroid drugs; RFA, radiofrequency ablation.

**Table 3:** Costs of RFA and surgery.

RFA		hEMItx		Short-stay	
Preprocedural costs					
Radiological visit	25.30	Surgical visit	25.30	Surgical visit	25.30
Laboratory tests	100.20	Laboratory tests	291.10	Laboratory tests	291.10
US	41.70	ECG	14.40	ECG	14.40
Otolaryngological visit	1.70	Chest X-ray	26.90	Chest X-ray	26.90
Anaesthesiological visit	34.50	Otolaryngological visit	34.50	Otolaryngological visit	34.50
		Anaesthesiological visit	34.50	Anaesthesiological visit	34.50
Procedural costs					
(A) Equipment					
Needle	1,240.70	Operating theatre	1,920.00	Operating theatre	1,980.00
US machine	3.00	Tools	197.60	Tools	197.60
Drugs	1.70	Drugs	664.00	Drugs	664.00
(B) Personnel					
Radiologist	57.00	Surgeon (×2)	202.60	Surgeon (×2)	207.60
Nurse (×2)	45.50	Theatre nurse	41.50	Theatre nurse	42.50
Anaesthetist	57.00	Nurse (×2)	82.90	Nurse (×2)	85.00
		Anaesthetist	101.30	Nurse (×2)	103.80
(C) Hospitalization					
Hospital day	—	>24 hours hospital stay	862.50	<24 hours hospital stay	375.00
Follow-up costs					
Laboratory tests	11.50	Laboratory tests	22.70	Laboratory tests	22.70
US	41.70	Otolaryngological visit	34.50	Otolaryngological visit	34.50

Total	€1661.50	Total	€4556.30	Total	€4139.40

All costs are expressed in euro. ECG, electrocardiogram; hEMItx, hemithyroidectomy; RFA, radiofrequency ablation; US, ultrasonography.
